# Digital Media Use and Psychosocial Health among Adolescent Boys and Young Men

**DOI:** 10.1007/s11920-026-01681-5

**Published:** 2026-05-14

**Authors:** Jason M. Nagata, Patrick Low, Eliot D. Lee, Selena Yu, Alicia W. Leong, Kevin Bao, Jennifer H. Wong, Megan A. Moreno, Jason M. Lavender

**Affiliations:** 1 Department of Pediatrics, University of California, San Francisco, 550 16th Street, 4th Floor, Box 0503, San Francisco, CA 94143 USA; 2 Department of Pediatrics, University of Wisconsin–Madison, Madison, WI USA; 3 Military Cardiovascular Outcomes Research Program (MiCOR), Department of Medicine, Uniformed Services University of the Health Sciences, Bethesda, MD USA; 4 The Metis Foundation, San Antonio, TX USA

**Keywords:** Media, Video games, Artificial intelligence, Male health, Adolescent boys, Young men

## Abstract

**Purpose of Review:**

To synthesize recent literature highlighting how and why adolescent boys and young men may face unique media-related psychosocial health risks and potential benefits.

**Recent Findings:**

Adolescent boys and young men appear to demonstrate distinct media use profiles, including higher engagement with video gaming, masculinity-oriented and appearance-focused social media content, online communities such as the manosphere, and, more recently, generative artificial intelligence tools.

**Summary:**

For adolescent boys and young men, gendered digital environments intersect with developmental processes related to identity formation, masculinity norms, social connection, and risk-taking, with important implications for mental health outcomes such as depression, anxiety, body dissatisfaction, muscle dysmorphia, disordered eating, aggression, and social isolation. Understanding these distinct media use profiles may allow clinicians, educators, and families to develop tailored approaches for screening, intervention, and prevention, supporting healthier development for adolescent boys and young men.

## Introduction

Digital media are now embedded across multiple domains of adolescent life, and a growing number of studies have focused on investigating digital media use in relation to adolescent mental health. However, the existing literature is characterized by substantial heterogeneity in findings, likely due to a number of conceptual, methodological, and experiential differences across investigations [1, 2]. As such, researchers have argued that understanding potential impacts of digital media use on adolescent mental health should focus on specific subgroups, given the potential for unique risk factors and use patterns across groups [3]. Notably, much of the research in this area has centered on adolescent girls and young women or has not adequately considered gender-specific patterns of engagement with digital media. Evidence indicates that adolescent boys and young men demonstrate distinct media use profiles, including higher engagement with content coded with traditionally masculine-associated traits (e.g., status, competition) [4, 5]. Moreover, the quantity, type, and content of media engagement may impact mental health concerns among adolescent boys and young men (e.g., depression, anxiety, body dysmorphia, disordered eating), which are routinely underrecognized [6–9].

Elucidating the relationship between digital media use and mental health in adolescent boys and young men is particularly important given evolving sociocultural trends that are reflected in digital media engagement. For example, online misogyny and the “manosphere,” which may package harmful gender narratives alongside adjacent interests (e.g., fitness, dating advice), have prompted calls for relevant research in this population [10–12]. The increasing ability of social media algorithms to deliver highly personalized content may heighten mental and behavioral health risk among those who are already the most vulnerable [13, 14]. Notably, the growing prevalence of generative artificial intelligence (AI) tools and their use among adolescents and young adults for seeking mental health advice has also raised questions about safety and the potential for exacerbating symptoms across multiple domains of physical and mental health [15, 16]. This narrative review synthesizes recent epidemiologic, experimental, and qualitative evidence that addresses media-related mental health risks for adolescent boys and young men, and offers suggestions for potential approaches to screening, prevention, and intervention.

## Gendered Patterns of Media Use and Associations with Mental Health

Recent research indicates that adolescent boys and girls differentially engage with digital media. In a cross-national analysis of adolescents aged 13–18 years old from the United States (U.S.) and the United Kingdom (U.K.), adolescent girls reported more time on smartphones, texting, and social media, whereas adolescent boys reported more time gaming. Specifically, adolescent boys spent 10 minutes more per day on gaming, and adolescent girls spent 38 minutes more per day on social media [17, 18]. Additionally, compared to adolescent girls, adolescent boys spent less time on visual and appearance-focused platforms and were less likely to engage in feedback-seeking behaviors [19].

Differential patterns of digital media engagement may contribute to gender differences in adolescent mental health risk. Associations with mental health are not uniform across digital activities; social media and general internet use are most strongly associated with self-harm behaviors, depressive symptoms, lower life satisfaction, and lower self-esteem than gaming or television viewing across adolescents, and these associations are generally stronger in adolescent girls than in adolescent boys [20]. More broadly, heavier digital media use is associated with lower psychological wellbeing in both genders, although these associations tend to be weaker in adolescent boys than in adolescent girls [17]. Light digital media use is associated with slightly greater wellbeing than no use, particularly among adolescent boys [17]. Activity-specific differences may be especially relevant when examining online gaming, an activity in which adolescent boys report greater engagement and that has been associated with lower positive wellbeing in both adolescent boys and girls [21]. Significant gender differences can exist even within particular media. For instance, one study found that young men preferred strategy games, whereas young women preferred brain and skill games [22]. Developmental timing may differ by gender as well, with negative associations between social media use and wellbeing emerging earlier in adolescent girls (around 11–13 years) than in adolescent boys (14–15 years) [19].

Patterns of problematic use also have been found to differ in adolescent boys relative to adolescent girls. In adolescent girls, emotional problems often precede both problematic social media use and problematic gaming [6]. In contrast, among adolescent boys, problematic gaming has been found to precede emotional problems, and hyperactivity and inattention precede problematic social media use [6]. Notably, general problematic internet use does not significantly differ between adolescent boys and girls [23, 24]. However, adolescent boys and men report higher problematic video game use, and adolescent girls and women report higher problematic social media and mobile phone use [25, 26]. Altogether, these findings highlight gender differences in digital media consumption, which clinicians and researchers may consider when assessing internet usage and targeted interventions.

## Social Media

### Masculinity Norms, the Manosphere, and Online Misogyny

Digital media serve as powerful socializing agents for masculine norms among adolescent boys and young men. Media representations of adolescent boys and men perpetuate restrictive gender portrayals, and exposure to stereotypical content can strengthen beliefs in gender stereotypes and traditional gender role norms [27]. The shift toward hypermuscular male imagery in media has likely contributed to internalization of unattainable body ideals, particularly among adolescents undergoing identity formation and heightened peer comparison [28]. Higher adherence to stereotypical male gender role norms, such as emotional restriction, is generally associated with higher internalizing behavior problems and lower social support [29]. Moreover, findings indicate that greater conformity to traditional masculinity during the middle school transition period is associated with greater depressive symptoms and decreased academic engagement for both adolescent boys and girls, with boys showing increased conformity and decreased help-seeking behaviors [30]. Furthermore, mental health risk may vary across specific masculine norms. For example, greater conformity to violent norms and self-reliance are associated with higher odds of suicidal ideation among adolescent boys and young men [31].

The “manosphere” refers to a constellation of online communities that espouse misogynistic and anti-feminist ideologies alongside seemingly benign adjacent interests such as fitness and dating advice, which may be easily accessed by young audiences [10]. Misogynistic content is often presented on social media as entertainment, promoting high engagement and increasing the potential for impacts on behavior, mental health, and peer relationships when consumed [13]. Despite notable engagement among young men, data specific to how manosphere content directly impacts adolescent boys and young men remain limited [10]. However, significant proportions of school teachers have reported high concern regarding the influence of online misogyny on students, including male students praising misogynistic influencers, making misogynistic comments, and engaging in discriminatory behavior toward female peers and staff [11]. These findings suggest that exposure to online misogyny and manosphere content may significantly affect adolescent boys’ behavior, psychosocial functioning, and identity development.

### Body Image, Muscularity-Oriented Content, and Muscle Dysmorphia/Eating Disorders

Adolescence and young adulthood are important developmental periods during which individuals form their identities and engage in heightened peer comparisons [32]. These processes are increasingly affected by social media use, which is among the most pervasive and impactful sociocultural influences among youth [33]. Social media can feature content and images highlighting an idealized masculine physique [33] and thus may play a potent role in fostering appearance comparisons and body dissatisfactions. Importantly, adaptive social media algorithms that display content based on engagement patterns can further intensify the relationship between social media use and dissatisfaction [34]. Frequency of social media use is associated with negative body image in both adolescent boys and girls, with specific behaviors (e.g., selfie editing) identified as particularly salient to dissatisfaction versus usage duration alone [35]. The impact of social media, in turn, is moderated by the degree of internalization of the thin or muscular body ideal. For example, among adolescent boys with higher internalization of either thin or muscular body ideals, greater social media use is associated with increased body dissatisfaction [36].

Of particular concern in adolescent boys’ and young men’s digital media use is the elevated risk of muscle dysmorphia (MD), a condition characterized by a preoccupation with a perceived lack of muscularity. In a cross-sectional study of Canadian adolescents and young adults, greater total recreational screen time and texting were associated with higher MD symptoms across both adolescent boys and girls [37]. However, higher social media use emerged as a distinct predictor of greater MD symptoms in adolescent boys and young men [37]. Beyond duration of use, the content of social media has also been shown to be important. For instance, viewing “fitspiration,” or social media content intended to inspire healthy lifestyles, has been associated with increased muscularity-related attitudes and behaviors among undergraduate men [38]. Another cross-sectional study of an international sample of adolescent boys and men further highlighted that viewing muscularity-oriented content, such as images of hypermuscular bodies, was strongly associated with probable MD, independent of total time spent on social media [7]. Furthermore, in a predominantly young adult male sample of individuals with MD, exposure to idealized muscular bodies through “fitspiration” content was found to promote social comparison and negative self-worth [39].

Social media use is also associated with eating disorder (ED) psychopathology across different gender-specific social media engagement patterns [40], with body dissatisfaction being identified as a risk factor in the development of EDs [41]. For instance, a cross-sectional study of Norwegian adolescent boys and girls found that ED psychopathology was significantly associated with perceived pressure from family, peers, romantic partners, and social media, although the link between social media pressure and ED psychopathology was stronger in adolescent girls [41]. Notably, ED psychopathology was associated with the internalization of body ideals, where adolescent girls reported higher scores on appearance ideals and adolescent boys reported higher scores on muscular ideals. A longitudinal study of early adolescent boys and girls similarly found that screen time and social media use were prospectively associated with ED symptoms at a two-year follow-up [42].

Within this framework, the drive for muscularity inherent in MD appears to share a complex, bidirectional relationship with disordered eating patterns. On one hand, adolescent boys and young men with probable anorexia nervosa or bulimia nervosa report significantly greater muscularity concerns than those without these conditions, suggesting a pursuit of muscularity may drive restrictive or compensatory eating behaviors [43]. Conversely, among German men, those at risk for MD showed a statistically significant 5.6% higher probability of engaging in binge-eating behaviors compared to those not at risk [44]. This interconnectedness between the pursuit of muscularity and changes in eating patterns is further amplified by digital influences, as social media addiction is significantly associated with ED symptoms, with MD symptom severity as a mediating factor [45].

### Supplements, Anabolic-Androgenic Steroids, and Risk-Taking

The internalization of muscular ideals and body dissatisfaction arising from digital media use may promote the use of appearance- and performance-enhancing drugs and substances (APEDS) [46]. In particular, the association between social media use and APEDS use is nuanced, as total time spent on social media is not always associated with all types of APEDS use. For instance, a cross-sectional study of young male gym users found that engagement with image-centric platforms, such as Instagram, and exposure to fitness-related content were associated with body dissatisfaction and the use of dietary supplements and anabolic-androgenic steroids (AAS). The sheer frequency of social media use, however, did not show similar associations [47]. Research involving Canadian adolescents and young adults found that while greater total social media use was associated with consumption of energy and pre-workout drinks, engagement with fitness- and weight-related content was associated with use of caffeine, creatine, probiotics, and whey protein [46]. Moreover, in a cross-sectional study of adolescent boys and men who had never used AAS, greater time spent on social media associated with higher intentions to use AAS, as were specific behaviors, including social media addiction, more frequent body comparisons on social media and greater viewing of muscularity-oriented content and muscle-building drugs and supplements [48].

Dangerous behavioral trends among adolescent boys and young men may also be promoted via digital media use. For example, “dry scooping,” or consumption of undiluted pre-workout powder that can cause cardiac effects from high caffeine concentrations, digestive issues, and psychosis, has been shown to be significantly more common among men (21.8%) and correlated strongly with high social media use and MD symptoms [45]. Another high-risk trend is “bone-smashing” on TikTok; in response to this practice of using a hammer to alter facial bone structures through repeated trauma, maxillofacial surgeons have raised concerns regarding risks of fractures, neurovascular injury, and facial deformity [50]. Furthermore, the online “looksmaxxing” community, under the guise of self-improvement and maximizing one’s appearance, has encouraged men to undergo substantial physical alterations, including invasive procedures such as leg-lengthening surgeries and rhinoplasties [51]. These behaviors illustrate a broader trend of increased risk-taking among adolescent boys and young men who engage in appearance-focused social media content.

## Video Games

Studies have examined both potential negative and positive qualities of video gaming among adolescent boys and young men. With respect to potential benefits, gaming offers adolescent boys a distinctive space to express their identity and explore gender norms. For instance, one adolescent described his preference for playing *Grand Theft Auto* as an extension of his emerging masculine identity, which was reflected in his behavior at both home and school and in his aspirations to become a policeman [52]. These gaming spaces are also where adolescent boys can encounter and negotiate intensified messaging about traditional masculine norms of success, competition, and performance [53]. The overall goals of winning and mastery are key motivators for many adolescent boys who play video games, as they confer status and recognition both within the game itself and among peers beyond the screen, which, in turn, can reinforce traditional gendered behavior and values of success and achievement [53]. However, gaming can also provide a space for counter-hegemonic gender performances where male players can temporarily step outside of their gender roles by adopting female avatars to either leverage stereotypes for strategic gain or subvert assumptions about the competence of female gamers [54].

​Beyond identity and gender role exploration, adolescent boys may find belonging and acceptance within gaming communities [55]. Nationally representative data from the U.S. indicate that the vast majority (94%) of adolescent boys who play games do so with others, with 55% reporting that gaming has positively impacted their friendships and 46% indicating that games have improved their ability to work with others [56]. Black adolescent boys, in particular, are also more likely to have interesting conversations and make new friends while gaming compared to their White or Latino peers [55]. The structured, task-focused nature of gaming also facilitates a more organic way for adolescent boys who encounter social challenges offline, particularly autistic adolescent boys, to communicate and develop friendships by reducing the burden of initiating conversation and alleviating social anxiety [57]. Furthermore, the sense of agency gained through these in-game avatars can translate into increased confidence, facilitating real-world social interactions with peers met online [58]. These findings suggest that video gaming may provide adolescent boys with a more natural and accessible platform for discussing nongaming topics compared to in-person interactions, especially for adolescent boys from marginalized communities [59].

While gaming can offer meaningful developmental and social opportunities, specific motivations and patterns of play among adolescent boys are also linked to an elevated risk for Internet Gaming Disorder (IGD). Male gender has been found to be an independent risk factor for IGD, even after adjusting for gaming time and individual characteristics [22, 24, 60]. One study of Chinese gamers found that young men are more likely to prefer strategy games [22], which are associated with greater IGD symptoms compared to other game genres [22, 61]. The intensity of play is another risk factor, with binge-gaming (playing for 5 or more consecutive hours) being more prevalent among young men and showing a dose-response relationship with IGD severity and poorer mental health outcomes, including lower life satisfaction and impaired sleep quality [63]. Furthermore, adolescent boys are more likely to game and communicate with strangers, a pathway that may reinforce problematic play and increase exposure to harmful conduct, such as harassment, which is often normalized as “trash talk” within competitive gaming environments [53, 64].

​These elevated risk patterns can impact the mental health of adolescent boys, as longitudinal research has established a reciprocal relationship between IGD and depression, with the cycle appearing more pronounced and rapidly escalating in adolescent boys compared to adolescent girls [65]. Gaming can temporarily relieve distress in adolescents, but may hinder healthier coping development, worsen depressive states, and foster over-reliance on gaming [65]. This over-reliance may be reinforced by heightened sensitivity to gaming-related rewards in men, sustaining IGD and impairing inhibitory control, making disengagement harder [66]. Moreover, this pattern is amplified with weak offline support systems. For instance, a study of Iranian adolescent boys with gaming disorder revealed that participants used in-game relationships to compensate for a lack of emotional connection at home [67]. Similarly, research on Chinese adolescents and young adults found that being an only child was a risk for problematic gaming, suggesting that family structure and parental attention may shape gaming habits [68].

## Generative AI

Generative AI tools are emerging as a new digital influence on psychological development among adolescent boys and young men. A U.S. national survey found that 53% of adolescent boys and young men have ever used generative AI, with 14% using it once or twice a week [69]. This pattern extends beyond the U.S., with surveys from the U.K. indicating that 87% of male internet users aged 16–24 have used a generative AI tool in the past year [70]. Adolescent boys and young men report predominantly using generative AI to gather information, brainstorm ideas, and write code, suggesting they may be using AI largely as a productivity tool [69].

Beyond frequency of use, adolescent boys and young men report greater confidence in using generative AI tools and are more likely to perceive AI-generated information as reliable [70, 71] compared to adolescent girls and young women. In a study of Australian adolescents and young adults, 74% of young men reported confidence in using AI tools, compared with 56% of young women, a gap that persisted across both high school and tertiary education settings [71]. Adolescent boys also appear to be less likely than adolescent girls to anticipate predominantly negative impacts from generative AI on their lives [69]. Research from Norway further supports this difference in perspective, with adolescent girls holding more negative attitudes about the characteristics of AI-generated text [72]. Overall, adolescent boys and young men are more likely to have a positive attitude towards AI. However, a cross-sectional survey of young men in Pakistan found that exposure to AI-generated imagery was linked to lower body image satisfaction, mediated by increased social comparison [73].

In contrast to generative AI, adolescent boys were slightly more likely than adolescent girls to report never having used an AI companion (i.e., a digital conversational agent designed for personalized, relationship-like interaction rather than primarily task-oriented assistance) [74]. However, among adolescent boys who use AI companions, they are significantly more likely than adolescent girls to say they use it for entertainment [74]. Notably, 4–6% of adolescent boys report using AI companions for emotional distress, with U.K. data showing a 300% rise in AI companion discussions among adolescent boys over one academic year [74–76]. The potential for this trend to be a positive development is supported by prior literature, which suggests AI companions may offer a safe, non-judgmental environment for mental health conversations, particularly for young men [77]. Furthermore, there is increasing interest in leveraging them as a preventive care tool and to bridge gaps between in-person therapy sessions [77, 78]. However, AI companions carry risks, as they often lack therapeutic training to provide accurate advice and cannot reliably monitor or assess suicide risk, self-harm, or other crises [77, 79]. Current AI chatbot models may reinforce user-provided statements, potentially strengthening maladaptive core beliefs and exacerbating mental health problems [80]. Furthermore, adolescents with pre-existing emotional problems may turn to AI companions for coping, promoting unhealthy attachment and dependence [81]. It is important to consider differences across AI platforms, as a cross-sectional study of Belgian adolescents investigating emotional reactions to Snapchat’s My AI Chatbot found that there were no significant gender differences in use, though younger adolescents were more likely to use My AI and reported greater positive emotions [82].

## Conclusions

### Clinical and Public Health Implications

Across digital domains, digital media engagement among adolescent boys and young men is characterized not only by time spent online but also by content, context, and developmental timing [19]. Clinicians should recognize that gendered patterns of digital media use, particularly content reinforcing restrictive masculinity norms and stereotyped portrayals, may confer unique risks for adolescent boys and young men [27]. When assessing media usage, clinicians should consider not just frequency and duration but also the specific behaviors adolescents engage in and the nature of content consumed. Accordingly, routine care can incorporate a brief, developmentally sensitive digital media history assessment that moves beyond screen time to also examine: (1) platforms used and typical content encountered (e.g., fitness “transformation” feeds, competitive gaming, manosphere-adjacent information); (2) algorithmic personalization and “rabbit hole” experiences; (3) the online social environment (e.g., group chats/servers/communities, peer and community norms, support versus ridicule); and (4) the functions digital media use serves (e.g., using media for connection/belonging, identity exploration, achievement/competition, mood regulation/coping, boredom relief, validation/status). This approach acknowledges the potential developmental benefits of digital media while also paying attention to factors that may be associated with psychosocial and behavioral risks [13, 19].

Adolescence and young adulthood represent important windows for prevention, as these developmental periods are characterized by actively forming identity, calibrating peer norms, and establishing habitual patterns of digital engagement. Public health efforts should extend beyond generic “screen time” messaging by integrating skills-based, gender responsive digital literacy into school curricula and community youth programming that target algorithmic awareness, parasocial influence, digital citizenship education, anti-harassment skills, and AI credibility [83, 84]. Health care providers can reinforce these skills with anticipatory guidance that normalizes conversations about online content, peer dynamics, and motives driving digital media use. Table 1 adapts the American Psychological Association’s adolescent social media recommendations to provide practice-oriented guidance adapted for clinicians working with adolescent boys and young men, and Table 2 outlines domain-specific clinical, public health, and research priorities across social media and muscularity-oriented content, gaming, and AI tools.

### Limitations

The review is limited by a literature base dominated by cross-sectional, observational studies that cannot establish causality or longitudinal directionality between digital media use and mental health outcomes in adolescent boys and young men [2, 3]. Much of the evidence is also based on self-reported data, which are associated with potential recall and social desirability biases and often fail to capture the content-level and algorithmically curated nature of exposure (e.g., what participants actually use, with whom they interact, and how platform features shape engagement). As a result, mechanisms linking specific digital experiences to outcomes such as MD symptoms, ED symptoms, risk-taking behaviors, and APEDS use remain incompletely characterized. Evidence is particularly sparse for rapidly evolving exposures that are shaped by platform algorithms and vary widely by user, including manosphere-related material and recommender-driven “rabbit hole” pathways. Similar gaps apply to generative AI and AI companion tools, where experimental, epidemiologic, and sex- and age-disaggregated studies remain scarce [16, 74, 79, 81]. Across studies, adolescents are often analyzed in aggregate without adequately accounting for developmental stage or social context, masking meaningful heterogeneity in exposure pathways and risk trajectories for adolescent boys and young men specifically.

### Future Directions

Future research should prioritize prospective, experimental, and quasi-experimental studies that capture content-level digital exposures, motives for use, interaction quality, and developmental change over time in adolescent boys and young men. Measurement should move beyond reliance on self-report through approaches such as passive sensing, privacy-preserving feed audits, and product-level safety evaluation. Across social media, gaming, and AI, research should clarify mechanisms of risk and resilience, report functional and implementation outcomes, and more consistently stratify analyses by sex, gender and developmental stage while including marginalized and neurodivergent adolescent boys and young men. Domain-specific priorities are summarized in Table 2.

**Table 1:** Recommendations for social media use among adolescent boys and young men, adapted from American Psychological Association (APA) recommendations on social media use in adolescence

APA recommendation (paraphrased)^1^	Recommendation adapted for adolescent boys and young men
1. Encourage adolescents to use social media in ways that foster social support, companionship, and healthy relationship-building.	**1. Identify and reinforce protective online use for adolescent boys and young men (connection**,** support**,** and prosocial communities).** Assess which platforms/spaces support connection with offline friends, shared interests, and constructive communities, and which spaces are organized around dominance, humiliation, or misogyny. Reinforce prosocial online behaviors (e.g., encouragement, teamwork, repair after conflict) and encourage shift toward prosocial contexts that strengthen real-world relationships and social support [53].
2. Guide families to align social media features and privacy settings with youths’ developmental capacities. Provide age-appropriate counseling about how adolescents’ online activity can be stored, shared, and tracked for commercial purposes.	**2. Optimize age-appropriate privacy/feature settings**,** and teach how platform data are collected**,** shared**,** and monetized.** Prioritize platform settings that reduce unwanted contact and limit high-salience engagement features that can amplify status- and appearance-based pressures (e.g., restrict comments/DMs from unknown accounts; tighten comment/reply permissions; limit recommender-driven “suggested” content; disable autoplay/endless scroll). Provide brief data use literacy counseling using salient examples (e.g., fitness/physique-focused content about bulking/cutting and supplement/steroid use; competitive gaming communities; and dating/manosphere-adjacent clips can shape recommendations and targeted advertising). Clarify that searches, DMs, and chats may be stored or shared (e.g., screenshots/forwarding), and discuss privacy expectations and safety planning when sensitive topics are involved given potential use of private digital spaces to explore sensitive topics (e.g., physique goals, sexuality/relationships, emotional distress) they may be less likely to discuss in person [13, 85].
3. For early adolescents (10–14 years), recommend a combined approach of caregiver discussion and boundary setting related to social media use. As digital literacy grows, increase autonomy gradually while balancing oversight with privacy and emphasizing caregiver role-modeling around device/social media use.	**3. Recommend structured**,** collaborative caregiver-youth feed check-ins and routines to support healthy use of social media.** Encourage periodic check-ins centered on what shows up on feeds and how mood/behavior are affected, paired with predictable routines (e.g., device-free meals, consistent bedtime phone location outside the bedroom). Emphasize caregiver role-modeling (e.g., minimize “phubbing” or phone use during conversations). Recommend low-pressure prompts to support open discussion of online experiences, given that distress or peer conflict may not be automatically disclosed [3, 13].
4. Assess for exposure to content that promotes self-harm, harm to others, or eating-disordered behavior, particularly whether recommendation algorithms are reinforcing this content. Counsel on concrete steps to reduce exposure (e.g., block/report, filters, feed resets), and complete risk assessment for self-harm/violence or medically unsafe behaviors when indicated.	**4. Assess exposure to high-risk content and intervene with exposure reduction steps and safety assessments.** Assess whether feeds repeatedly surface concerning content, such as self-harm/suicide, violence/aggression, humiliation “challenge,” or extreme fitness/physique (e.g., unsafe supplement “stacks,” steroid use, severe restriction, purging tips, excessive exercise, “cutting/bulking” extremes). Offer concrete steps to reduce exposure (e.g., block/report, content filters, reset recommendations, disable autoplay), and complete standard risk assessment when exposure is linked to current suicidal ideation, threats, or unsafe behaviors, recognizing that online reinforcement may increase risk [34, 42].
5. Assess for online peer victimization (cyberbullying), identity-targeted harassment, and exposure to hateful or extremist content (cyberhate). Support adolescents and caregivers in recognizing and critically evaluating discriminatory messaging. Provide guidance on coping and help-seeking strategies (e.g., block/report, document when needed, coordinate with school supports).	**5. Assess cyberbullying/cyberhate experiences and implement a concrete coping**,** reporting**,** and school support plan.** Assess online peer victimization and exposure to cyberhate, including contexts where aggression is normalized (e.g., group chats/servers with dogpiling, exclusion, misogynistic slurs). Evaluate mental health sequelae (e.g., shame, anger/irritability, withdrawal, school avoidance) and develop an actionable response plan: safety steps (block/report, documentation), coping strategies, and coordination with school/caregiver supports when needed. Include brief skill-building to help recognize discriminatory messaging and practice safety strategies (e.g., disengage, support targets, seek adult help) especially as online harassment may be interpreted as “normal” and lead to underreporting of distress [13, 53].
6. Routinely screen for problematic social media use by assessing loss of control, functional impairment, and whether use is crowding out or helping avoid in-person interactions. Use screening results to set specific targets (e.g., sleep protection, building offline coping and social routines), treat comorbid symptoms, and involve caregivers when appropriate.	**6. Evaluate problematic social media use beyond time-based metrics alone and set 1**–**2 measurable treatment targets.** Assess for sleep/school/relationship disruption, compulsive features (distress/irritability when interrupted, secrecy/deception to maintain access), and relevant content-specific compulsions (e.g., recurrent physique checking/comparison, repeated engagement with misogyny-oriented content). Use screening results to set measurable targets (e.g., consistent bedtime routine that limits social media use, replace device-based avoidance with alternative offline coping activities, schedule regular in-person connection) and address comorbidity (e.g., depression, anxiety, ADHD). As appropriate, involve caregivers to support routines, reduce exposure to hostile peer spaces, and reinforce offline coping and connection. This focus is clinically important, because problematic use may present as irritability, withdrawal, or risk-taking [2, 19].
7. Advise families to protect sleep and physical activity by setting social media boundaries, prioritizing regular sleep-wake schedules and minimizing social media use before bed.	**7. Prioritize sleep and physical activity as core clinical targets**,** especially for those whose evening routines combine social media with gaming or group chats.** Counsel families to implement concrete boundaries that protect ≥ 8 h of sleep and a regular schedule (e.g., no social media in the hour before bed; devices charge outside the bedroom; “bedtime mode”), and to plan daily physical activity as a mood and stress regulation strategy. Frame sleep and physical activity as relevant to mood regulation: sleep disruption may present as irritability or behavioral dysregulation, while physical activity can support mental health [3].
8. Assess appearance-based social comparison and photo/feedback-focused behaviors (e.g., frequent checking, editing, distress tied to likes/comments). Counsel on strategies to reduce comparison-driven distress (e.g., limiting triggering accounts, strengthening offline sources of self-worth).	**8. Assess physique-related body image concerns**,** including appearance comparison**,** validation seeking**,** and compulsive checking.** Inquire about exposure to lean/muscular ideals, time spent checking one’s physique (mirror and photo checking), photo/feedback cycles (posting/editing for validation; distress tied to likes/comments), and downstream behaviors (e.g., restrictive dieting, compulsive exercise, supplement escalation, AAS interest/use). Evaluate associated impairment and comorbidity (depression/anxiety, irritability, social withdrawal). Screen for red flags, such as training despite injury, rapid dietary restriction, or concealment of supplement/AAS use. Consider interventions such as content curation and psychoeducation about manipulated imagery, CBT-informed targets for comparison and checking behaviors, values-based fitness goals, and referral to specialty eating disorder/body image care when muscle dysmorphia/eating disorder symptoms or medical risk are present [7, 28, 36, 43, 48].
9. Integrate social media literacy skill-building into treatment and anticipatory guidance. This includes credibility checking, recognition of mis/disinformation tactics, and correction of distorted inferences about peer norms, alongside conflict resolution and safe mental health communication.	**9. Integrate digital literacy into care in ways that address how online norms are encountered and interpreted.** Improve understanding that suggested content is shaped by engagement signals and may disproportionately amplify provocative content (e.g., humiliation-based “status” content, misogynistic narratives, extreme fitness advice). Teach practical skills for evaluating credibility and intent, including identifying misinformation tactics, checking sources for health claims, and identifying distorted normative inferences (assuming viral content reflects typical peer beliefs/behavior). When relevant, discuss manosphere-adjacent messages and identify alternative narratives that support respect, empathy, and prosocial relationship skills [10, 13].
10. Support sustained investment in research that includes long-term longitudinal studies, younger age groups, and marginalized populations. Advocate for independent researchers’ access to platform data to evaluate social media’s effects and guide targeted prevention and intervention.	**10. Support a clinically-actionable evidence base for mental health among adolescent boys and men in the digital age by incorporating digital exposure/function and outcome tracking into routine care and referral pathways.** Routinely document key digital exposures and functions alongside symptoms (e.g., physique-focused comparison, harassment/toxicity exposure, and avoidance- or status-driven use), and tracking functional outcomes (e.g., sleep, academic functioning, social withdrawal) over follow-up. At the systems level, incorporate concise prompts in intake templates and standardize referral pathways for high-risk presentations (e.g., severe cybervictimization, gaming disorder, muscle dysmorphia/eating disorder symptoms). Clinicians and professional organizations should also support research that disaggregates findings by sex/gender and developmental stage and includes marginalized adolescent boys and young men [2, 3, 19].

^1^Recommendations in the left column are paraphrased from the American Psychological Association’s 2023 Health Advisory on Social Media Use in Adolescence and adapted in wording for clinical use [86]

^2^Adaptations specific for adolescent boys and young men in the right column are informed by the literature reviewed in this article

**Table 2:** Clinical, public health, and research priorities across social media, gaming, and artificial intelligence (AI) tools for adolescent boys and young men

Domain	Clinical implications and priorities	Public health implications and priorities	Future research directions
Social media (physique-focused content; manosphere-adjacent exposure)	**Assessment**: • ***Clarify function***: connection/support, avoidance, validation/status • ***Identify content-level exposures***: physique-focused, bulk/cut, transformation content, supplement/steroid marketing, misogyny/manosphere-adjacent narratives, harassment/humiliation-based peer environments [7, 10, 13, 48, 86] **Signs of clinically concerning use**: • ***Distress may present as irritability***,*** shame***,*** withdrawal***,*** or risk-taking***; may underreport distress due to self-reliance norms [3, 19, 27] • ***Body image-related warning signs***: repetitive physique comparison/checking, rigid dieting or compulsive training, escalating or concealed supplement/steroid interest or use [42, 43, 48] **Prevention and intervention**: • ***Reduce exposure to harmful content***: block/report, content filters, feed resets, disable autoplay [34, 86] • ***Shift toward prosocial/supportive spaces and reduce engagement with humiliation-based spaces*** [13, 53] • ***Refer/triage when body image risk is clinically significant***: includes probable muscle dysmorphia/eating disorder symptoms or medically risky eating/exercise/supplement use behaviors [7, 42, 43]	• ***Algorithmically amplified exposure may reinforce harmful gender norms during identity formation***: Recommender systems can increase exposure to misogynistic, harassment-prone, and hypermuscular or physique-focused content, reinforcing restrictive masculinity norms and intensifying body dissatisfaction during a developmentally sensitive period [7, 13, 27] • ***Commercialized body image content may normalize and valorize risky appearance-enhancing behaviors***: Idealized physique content is often paired with influencer- and supplier-driven promotion of supplements, steroids, and transformation-oriented products or trends, making rapid body change appear desirable, attainable, and socially rewarded for younger users [7, 48, 87–89] • ***Prioritize strengths-based digital literacy***,*** platform accountability***,*** and non-stigmatizing harm reduction outreach***: Public health efforts should support adaptive uses of social media for connection and identity exploration, while aiding recognition of manipulated imagery, commercial persuasion, and risky appearance enhancement content. Responses should also avoid purely punitive framing and expand access to credible, non-stigmatizing harm reduction information for youth already encountering appearance- and performance-enhancing substances [83, 84, 87, 90]	• ***Move beyond time-based metrics to content-level and algorithmic exposure measurement***: Future studies should quantify specific social media exposures relevant to adolescent boys and young men, including hypermuscular imagery, supplement/steroid marketing, manosphere-adjacent content, and recommender-driven “rabbit hole” pathways, using repeated measures, passive sensing, or privacy-preserving feed audits linked to mental health outcomes [7, 13, 19, 42, 48] • ***Clarify mechanisms and developmental heterogeneity***: Research should test how restrictive masculinity norms, body-ideal internalization, social comparison, peer dynamics, and motives for social media use shape risk across developmental periods, and whether effects differ by baseline psychopathology, offline support, and marginalized identities [7, 10, 19, 27] • ***Evaluate intervention effectiveness***,*** acceptability***,*** and equity of reach***: Priorities include randomized or quasi-experimental evaluations of gender-responsive digital literacy, platform and policy changes, and non-stigmatizing harm reduction approaches. Attention should be paid to which messages and supports are acceptable and effective for adolescent boys and young men already engaging in or considering appearance-enhancing practices, particularly when these practices are tied to self-worth or social validation [83, 84, 87, 89, 90]
Gaming (social play; harassment; internet gaming disorder)	**Assessment**: • ***Clarify function***: achievement, competition, belonging, emotional relief and escapism [53, 64] • ***Differentiate normative play from gaming disorder***: assess functional impairment, loss of control, and continued use despite harms [65, 91] • ***Assess comorbidity*** (depression/anxiety, ADHD) and ***sleep disruption*** [65, 91] **Signs of clinically concerning use**: • ***Functional deterioration***: worsening sleep, declining school performance, reduced participation in offline activities and relationships [65, 91] • ***Loss of control***: irritability or distress when interrupted, repeated failed attempts to cut down, continued play despite harms [91] • ***Concealment or avoidance***: secrecy or deception about gaming time or content; gaming used primarily to avoid distress or responsibilities [65, 91] **Prevention and intervention**: • ***Use CBT-informed strategies when impairment is present***: reduce gaming-as-avoidance and challenge gaming-related maladaptive beliefs (self-worth contingent on in-game rank/status; gaming as necessary for social acceptance; being offline will lead to peer rejection), while building alternative offline coping and reward sources that improve mood, self-confidence, and connection [92, 93] • ***Protect sleep and create predictable routines that reduce late-night gaming and dysregulation***: consistent bedtime routine, planned end-of-play time, changing device availability/location at night [92, 93] • ***Support safer online participation by helping caregivers customize/structure adolescent boys' and young men's gaming context***: encourage gaming with known peers or on moderated servers; apply default privacy settings that restrict data collection and unsolicited friend requests and messaging [53, 94]	• ***Gaming can support belonging and competence building***,*** and identity exploration***,*** but some competitive spaces also normalize status-seeking through humiliation and harassment***: Gaming can offer accessible peer connection, teamwork, and confidence building. However, some gaming environments also reward peer validation through humiliation-based “trash talk,” exclusion, and other hostile interactions [52, 53, 55–57] • ***Gaming may become more problematic when used as a primary strategy to manage distress***,*** especially when play is high intensity or occurs in less accountable online spaces***: Binge gaming and reliance on gaming for relief from distress are warning patterns associated with poorer sleep and wellbeing. Depressive symptoms and problematic gaming may also reinforce one another over time, particularly when offline supports are weak. Gaming and communicating with strangers may increase risk for less accountable, harassment-prone interactions that sustain problematic engagement [62–65, 67] • ***Prioritize safer social play and platform standards that preserve gaming’s relational benefits***: Public health efforts can expand access to cooperative and moderated gaming spaces, help families and schools identify when gaming shifts from social connection to sleep/coping displacement, and require stronger reporting, moderation, and age-appropriate communication safeguards in harassment-prone environments [53, 55, 57, 95]	• ***Identify which interaction and platform features shift gaming toward social connection versus problematic engagement for adolescent boys and young men***: Future studies should compare cooperative/friend-based and moderated gaming spaces with high-intensity, stranger-heavy, or harassment-prone environments. Studies should test how communication norms, moderation practices, and platform features shape belonging, confidence, and social-emotional learning versus problematic engagement [53, 55, 57, 95] • ***Clarify which patterns of gaming***,*** coping with distress***,*** and social contexts sustain gaming-related impairment over time***: Longitudinal studies with repeated measures should evaluate how high-intensity gaming, distress-driven overreliance on gaming, sleep disruption, weak offline supports, and weakly moderated interaction settings contribute to loss of control, functional disruption, and worsening wellbeing over time. This work should also further characterize the temporal and reciprocal relationship between depressive symptoms and problematic gaming [62–65, 67] • ***Determine which interventions reduce gaming-related harms without undermining gaming’s relational benefits for adolescent boys and young men***: Priorities include trials that match treatments to gaming motives and comorbidities and evaluate whether platform moderation tools, communication settings, and supportive community features reduce harassment while preserving connection, teamwork, and belonging. Implementation outcomes (feasibility, acceptability, uptake, and equity of reach) and functional outcomes (sleep, school functioning, offline relationships, wellbeing) should be reported alongside problematic gaming and relevant mental health symptoms [53, 92, 93, 95, 96]
Artificial intelligence tools (generative AI; AI companions)	**Assessment**: • ***Clarify function***: task support, learning, and exercising creativity (information seeking, planning, brainstorming, storytelling) versus reassurance-seeking, companionship, or avoidance [69, 74, 97] • ***Ask about higher-risk use***: seeking guidance on health and body image; sharing identifying or sensitive information [79, 97] • ***Assess displacement***: assess whether AI use is replacing sleep and offline supports and problem-solving, help-seeking coping skills [69, 74,79] **Signs of clinically concerning use**: • ***Preferential reliance on AI as primary confidant***,*** with limited offline disclosure***: AI is used as the primary outlet for sensitive concerns (distress, performance pressures, body image concerns, relationships and sexuality), especially in the context of minimal disclosure to peers and trusted adults [74, 85, 97] • ***Reassurance-seeking/compulsive use when distressed***: engagement in repetitive “checking” or reassurance queries with AI, with escalating time spent and increasing difficulty disengaging [79, 81] • ***Use in high-risk context and following clinically unsafe guidance***: AI use during acute distress (self-harm urges, suicidal ideation), or following AI-generated clinical guidance that is inaccurate, unsafe, or discourages appropriate help-seeking [79, 81] **Prevention and intervention**: • ***Provide structured AI guidance***: frame outputs from AI tools as non-authoritative; encourage verification of health-related claims using trusted sources; set clear privacy boundaries (avoid sharing identifying or sensitive details); reinforce using AI as an adjunct rather than substitute for clinical care [79, 97] • ***If AI is used for emotional support or reassurance seeking***: Screen for acute risk when indicated (self-harm urges or suicidal ideation), clarify function (loneliness, anxiety, avoidance), and develop a plan to strengthen offline supports and coping strategies (identify a trusted contact, outline steps during distress, and reduce reassurance loops) [79, 81] • ***If AI is used for health guidance***: redirect to evidence-based resources and address underlying clinical concern (e.g., body image distress, anxiety, depressive symptoms), with referral when clinically indicated [79, 97]	• ***Trust in AI-generated information may heighten susceptibility to misleading or harmful outputs***: Adolescent boys and young men report greater confidence in using generative AI and are more likely to perceive AI-generated information as reliable. This may increase vulnerability to inaccurate health or mental health guidance, and in some cases, to body image harms linked to AI-generated imagery and social comparison [69–71, 73] • ***AI companions may offer privately accessed***,*** low-barrier***,*** and nonjudgmental social support but be unreliable in acute crisis***: Some adolescent boys and young men may turn to AI companions during emotional distress. These tools may feel nonjudgmental, immediately accessible, and more private, but current evidence suggests they may lack the therapeutic training and oversight needed to provide accurate guidance, may miss self-harm or suicidality cues, and may promote unhealthy attachment or dependence in emotionally vulnerable youth [74, 77, 79, 81] • ***Prioritize AI-specific literacy***,*** visible bot disclosures***,*** and crisis-responsive youth safeguards***: Public health efforts should help adolescent boys and young men and their families verify AI-generated health information, recognize when AI is replacing rather than supplementing offline support, and avoid sharing sensitive personal information. Youth-facing AI tools should provide regular plain-language reminders that users are interacting with AI and implement reliable crisis-routing or escalating protocols when self-harm, suicidality, or other acute safety risks are present [69, 70, 74, 79]	• ***Clarify which patterns of AI use support versus undermine wellbeing in adolescent boys and young men***: Future studies should distinguish potentially adaptive AI use for task support, learning, and creativity from potentially higher risk use for reassurance-seeking, body image guidance, and emotional reliance. Studies should test when AI supplements versus displaces offline support, help-seeking, and coping. Research priorities include sex/gender-disaggregated analyses and involvement of neurodivergent and racially/ethnically marginalized adolescent boys and young men [69, 71, 73, 74] • ***Move beyond cross-sectional self-reports to longitudinal***,*** experimental***,*** and product-level safety studies***: Longitudinal studies with repeated measures, experimental designs, and product audits should evaluate how trust in AI-generated information, exposure to AI-generated imagery, and use of AI companion apps shape mental health trajectories, help-seeking, body image, and unhealthy reliance over time in adolescent boys and young men. This work should also test how these effects vary by developmental stage, platform type, baseline vulnerability, and marginalized identity [71, 73, 74, 79, 81] • ***Determine which youth safeguards and guidance reduce harm without undermining adaptive AI use for*** **adolescent boys and young men**: Priorities include evaluations of visible bot disclosures, verification prompts for health information, safeguards around sharing sensitive personal information, and crisis-routing or escalation protocols for self-harm, suicidality, or other acute safety concerns. Studies should also test how clinician and caregiver guidance on AI use affects how adolescent boys and young men integrate AI into learning, coping, and help-seeking without displacing offline support [69, 70, 74, 79]

## Key References

- Peng P, Chen Z, Ren S, He Y, Li J, Liao A, et al. Sex difference in the longitudinal association between depressive symptoms and internet gaming disorder among Chinese adolescents: an explanatory analysis at the aggregate and symptom level. Addictive Behaviors. 2026;172:108499. 10.1016/j.addbeh.2025.108499.
  - ○ Explores the gendered differences in the longitudinal relationship between IGD and depression among Chinese adolescents.
- Waechter N, Meschik M. Peer socialization of adolescent boys in digital games: achievement, competition, and harassment. Communications. De Gruyter Mouton; 2023;48:457–81. 10.1515/commun-2021-0079.
  - ○ Examines the key motivations and social experiences of adolescent boys in video gaming.
- Tufail R, Shahwani AM, Khan W, Badar Y. Examining the impact of AI-generated content on self-esteem and body image through social comparison. Bulletin of Business and Economics (BBE). 2024;13:413–21. 10.61506/01.00514.
  - ○ Assesses the impact of AI-generated content on self-esteem and body image satisfaction among young adults.
- Chu J, Ganson KT, Testa A, Al-Shoaibi AAA, Jackson DB, Rodgers RF, et al. Screen time, problematic screen use, and eating disorder symptoms among early adolescents: findings from the Adolescent Brain Cognitive Development (ABCD) Study. Eat Weight Disord. 2024;29:57. 10.1007/s40519-024-01685-1.
  - ○ This longitudinal prospective study examines the associations between baseline social media engagement and eating disorder symptoms at a two-year follow-up, addressing a gap in the literature that has historically relied on cross-sectional observations.
- Ganson KT, Piatkowski T, Testa A, He J, Nagata JM. Social media engagement and anabolic-androgenic steroid use intentions among boys and men in Canada and the United States. Body Image. 2026;57:102057. 10.1016/j.bodyim.2026.102057.
  - ○ Beyond aggregate time spent on social media, a factor established in prior research, this study highlights that specific social media content and engagement patterns are additional important factors significantly associated with intentions to use AAS.
- Ganson KT, Testa A, Rodgers RF, Nagata JM. Associations between muscularity-oriented social media content and muscle dysmorphia among boys and men. Body Image. 2025;53:101903. 10.1016/j.bodyim.2025.101903.
  - ○ Extending beyond prior research on an individual’s muscularity-oriented attitudes and behaviors, this study examines various forms of muscularity-oriented social media content to determine their respective associations with probable muscle dysmorphia.
- Singh B, Zhou M, Curtis R, Maher C, Dumuid D. Social media use and well-being across adolescent development. JAMA Pediatr. 2026;e255619. 10.1001/jamapediatrics.2025.5619.
  - ○ Analyzes differential digital media use patterns and mental health outcomes in a large, longitudinal cohort of adolescents.
- Regehr K, Shaughnessy C, Zhao M, Cambazoglu I, Turner A, Shaughnessy N. Normalizing toxicity: the role of recommender algorithms for young people’s mental health and social wellbeing. Front Psychol. 2025;16:1523649. 10.3389/fpsyg.2025.1523649.
  - ○ Authors highlight growing prevalence and normalization of misogynistic content on social media platforms and its link to behavioral issues in young boys.
- Hartselle S, Deol Y, Miselis K. Media representation of boys. Child Adolesc Psychiatr Clin N Am. 2025;34:683–92. 10.1016/j.chc.2025.05.011.
  - ○ This article explores the impact of media on the development of traditional gender roles and masculinity in adolescent boys, emphasizing the prevalence of hypermasculine and heteronormative stereotypes.

## Data Availability

No datasets were generated or analysed during the current study.
